# Comparison of online adaptation strategies for magnetic resonance guided prostate radiation therapy

**DOI:** 10.1016/j.phro.2025.100816

**Published:** 2025-07-25

**Authors:** Iymad R. Mansour, Chris D. Johnstone, Victor Malkov, Daniel Létourneau, Peter Chung, Tony Tadic, Jeff D. Winter

**Affiliations:** a Radiation Medicine Program, Princess Margaret Cancer Centre, Ontario, Canada; b Department of Radiation Oncology, University of Toronto, Ontario, Canada

**Keywords:** Adaptive therapy, MR-Linac, Adapt-to-position (ATP), Adapt-to-shape (ATS), Accumulated dose

## Abstract

**Background and purpose:**

MR-guided adaptive radiation therapy allows for daily plan adaptation to account for anatomical changes. Two common strategies are adapt-to-position (ATP), involving re-optimization based on isocenter shifts, and adapt-to-shape (ATS), involving full recontouring and reoptimization. This study provides a dosimetric comparison of ATP and ATS using accumulated dose.

**Materials and methods:**

Dose accumulation was performed for 35 patients with prostate cancer treated on a 1.5 T MR-Linac. All patients received ATS-based treatment with either 30.0 Gy in 5 fractions (30.0/5) or 42.7 Gy in 7 fractions (42.7/7), using a 5 mm isotropic PTV margin. ATP plans were retrospectively simulated. For each fraction, dose was mapped to a reference image using deformable image registration and summed across fractions. Fractional and accumulated dose-volume histogram (DVH) metrics were compared between ATS and ATP and correlated with daily anatomical variation.

**Results:**

ATP and ATS achieved equivalent accumulated CTV D95 and D98 for both regimens. In the 30.0/5 cohort, small but statistically significant differences in OAR dose were observed: accumulated bladder D40 was 4 % lower for ATP (1.27 Gy; p = 0.0004), and rectum D50 was 1 % lower for ATP (0.40 Gy; p = 0.0008). Differences in rectum D1cc and bladder D5cc were not significant. In the 42.7/7 cohort, femur D5 was 3 % higher for ATP (0.83 Gy; p = 0.02); other differences were insignificant. Dosimetric differences across strategies correlated with interfraction motion.

**Conclusion:**

ATP and ATS provided equivalent target coverage. OAR differences were statistically significant in some cases but remained within clinical tolerances, suggesting minimal clinical impact.

## Graphical abstract

**Figure:**
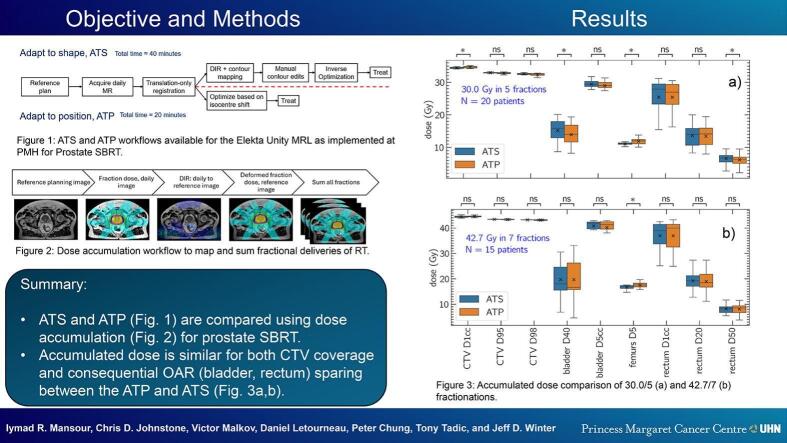

## 1 Introduction

Integrated magnetic resonance linear accelerator (MRL) systems have enabled the development of online adaptive strategies leveraging the improved soft tissue contrast afforded by MR images acquired at the time of radiation delivery [[1], [2], [3]]. These systems support multiple adaptation approaches, including Adapt-to-Position (ATP), which is based on a static model of the patient geometry (no deformation) and adapts treatment plans based solely on daily variations in the isocentre location, and Adapt-to-Shape (ATS), which adapts plans based on daily recontoured patient anatomy with full inverse optimization. ATS is considered more rigorous by adapting to individual anatomical variations throughout treatment including rotations and deformations. However, recent literature has demonstrated that ATP and ATS workflows exhibited dosimetrically comparable fractional dose distributions [4]. An ATS workflow increases the clinical burden (longer treatment times, clinical staff expertise) and should have clear clinical benefits to justify its use [4,5].

One approach to investigate different online adaptive workflows is to perform a quantitative analysis of both fractional (daily plan) dose and cumulative dose over the course of treatment. Analysis of fractional dose facilitates an evaluation of plan quality across adaptive workflows and the relationship of plan quality to daily anatomical variations, whereas dose accumulation using deformable image registration (DIR) yields an understanding of whether daily differences in adaptive strategies amount to systematic benefits over the complete course of treatment for individual patients and ultimately across groups of patients [[6], [7], [8]]. Although several studies have investigated prostate MRL treatment delivery using dose accumulation, no direct comparison of ATP and ATS workflows exist [4,5,[9], [10], [11], [12], [13]]. Furthermore, an understanding of how interfraction anatomical variations leads to differences in delivered dose from online adaptation has not been established.

This study compares ATP and ATS online adaptive radiation therapy (ART) strategies using DIR-based dose accumulation, and investigates how anatomical factors (*e.g.,* interfraction organ motion) contributes to dosimetric differences, with the aim of identifying patient features that influence plan quality and opportunities to further optimize adaptive treatment.

## 2 Methods

### 2.1 Patients

This retrospective study analyzed 35 patients with localized prostate cancer treated with ultra-hypofractionated MR-guided adaptive radiotherapy on an integrated 1.5 T MRI and 7 MV FFF linear accelerator, the Elekta Unity (Elekta Solutions AB, Stockholm, Sweden). The patient cohort included two fractionations:

• 30.0/5: 30.0 Gy in 5 fractions with 110 % dose to the clinical target volume (CTV), with subsequent 15 Gy single fraction high dose rate brachytherapy to the dominant prostatic lesion (N = 20 patients) or
• 42.7/7: 42.7 Gy in 7 fractions with 100 % dose to CTV (N = 15 patients).

All patients were treated on prospective clinical trials approved by our institutions research ethics board, with 30.0/5 patients treated on the ClinicalTrials.gov ID NCT04135794 study and 42.7/7 patients treated on NCT00913939. Study participation included informed written consent.

### 2.2 Treatment planning and online adaption

Both bladder and bowel preparation were required for all patients, including asking patients to have a bowel movement prior to simulation and treatment as well as drinking 300 mL at the start of each treatment session. Reference MR images were acquired using a 6-minute 3D T_2_-weighted sequence on the Elekta Unity system. Bulk density overrides were applied using the mean electron density from the patient body, femurs, and PTV contoured on a separated computed tomography (CT) simulation image (Canon Aquilion CT, Canon Medical Systems, Otawara, Japan). The CTV was defined as the prostate gland, with up to 1 cm of proximal seminal vesicles included at the treating physician’s discretion based on clinical factors, while the PTV was an isotropic 5 mm expansion of the CTV.

Reference treatment plans using 9-field IMRT were created in Monaco 5.40.01 (Elekta, Stockholm, Sweden) using Monte Carlo (MC) dose calculation incorporating magnetic field effects, 3 mm dose grid and 1 % statistical dose uncertainty. Clinical goals are provided in Supplemental Table S1. All patients were treated using ATS. At each daily fraction, a 2-minute 3D T_2_-weighted *localization MR* is acquired for online contouring and daily adaptation, followed by a repeat *verification MR* to assess if the target moved during plan adaptation.

#### 2.2.1 Adapt-to-shape (ATS)

The ATS workflow (Supplemental Fig. S1) adapts the reference treatment plan based on the patient’s anatomy at each fraction [1,14]. During the online planning session, the localization image was automatically rigidly registered to the planning MR image, followed by soft tissue translation matching by the radiation therapist. Initial reference contours were mapped from the planning to localization MR via deformable image registration (DIR) in Monaco, followed by contour editing by a radiation oncologist or fellow. The ATS optimization used the “*optimizing shapes and weights”* option for full inverse optimization starting from fluence with manually adjusted optimization objectives adapted from the reference plan.

#### 2.2.2 Adapt-to-position (ATP)

In contrast to ATS, the ATP workflow creates an adapted plan without the requirement of daily recontouring. Rather, the daily plan is reoptimized on the reference MR using the reference contours and rigid registration between the localization and reference images acquired during the online planning session. This effectively compensates for daily translational variations in patient setup, but since the reference patient geometry is used for optimization, it is not possible to account for daily changes in the shape and relative location of the targets and organs-at-risk. The ATP workflow was retrospectively simulated for all patients based on the localization MR using the “*optimizing shapes and weights”* option in Monaco.

### 2.3 Analysis

All reference, localization images, contours, and plans were imported into RayStation 10.1.1 (RaySearch Laboratories AB, Stockholm, Sweden) for fractional and accumulated analysis. ATS and ATP workflows were compared for both individual fractions and total accumulated dose using the identical set of online contours (sec. 2.3.1); evaluation included DVH (sec. 2.3.2) and conformity metrics (sec. 2.3.3). Changes in patient anatomy were quantified and related to dosimetric differences between ATS and ATP for individual fractions (sec. 2.3.4).

#### 2.3.1 Dose accumulation

**Fig. 1:**
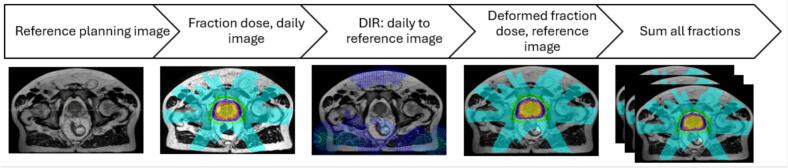
Dose accumulation workflow using deformable image registration (DIR) to map fractional doses to the reference MR; all mapped fractions are summed to compute the accumulated dose.

The dose accumulation workflow is presented in Fig. 1. Fractional ATP and ATS dose distributions on the localization MR were mapped to the reference image using DIR and then summed across all fractions to generate estimates of cumulative delivered dose. DIR used a hybrid image-intensity and structure-based algorithm with controlling regions of interest (CTV, rectum and bladder) and points of interest (POI) [15,16]. A geometric and dosimetric validation of the DIR strategy employed is described by Malkov et al., [17,18]. Cited work included the 30.0/5 patient cohort presented herein, with DIR accuracy assessed by comparing geometric agreement and DVH differences between propagated and expert-defined manual contours. The mean distance to agreement was 0.012 ± 0.001 cm for CTV, 0.020 ± 0.002 for bladder and 0.019 ± 0.002 cm for rectum ROIs. The DIR dosimetric accuracy was evaluated by calculating DVH differences between propagated and manual contours which yielded −0.036 ± 0.058 Gy (D98%, clinical goal: >28.5 Gy) for CTV, 0.02 ± 0.16 Gy (D5cc, clinical goal: <30.0 Gy) for bladder, and 0.05 ± 0.24 Gy (D1cc, clinical goal: <30.0 Gy) for rectum.

#### 2.3.2 Dose volume histogram evaluation criteria

Table 1 presents DVH criteria used for evaluating fractional and accumulated dose distributions. The PTV reference planning goals were applied to the CTV for accumulated dose evaluation. Organs at Risk (OARs) were limited to those structures that were routinely recontoured in the online sessions.

**Table 1:** Dose volume histogram (DVH) region of interest (ROI) evaluation criteria used for both 30.0/5 and 42.7/7 patients respectively. Note that we report fractional dose scaled up by the number of fractions to support use of the same evaluation criteria for fractional and cumulative doses.

ROI	Metric	30.0/5 (Gy)	42.7/7 (Gy)
CTV	D98	>28.5	>40.6
	D95	>30.0	>40.6
	D2cc	<35.0	<46.1
Rectum	D1cc	<30.0	<42.7
	D20	<20.0	<24.0
	D50	<10.0	<10.0
Bladder	D5cc	<30.0	<42.7
	D40	<15.0	<18.0
Femur	D5	<12.0	<18.0

#### 2.3.3 Dose conformity

Dose conformity, *C*, was calculated for the CTV and PTV using:

(1) $$C=\frac{V_{\mathrm{ID}}^{\mathrm{in}}}{V_{\mathrm{ID}}^{\mathrm{total}}}$$

where $V_{\mathrm{ID}}^{\mathrm{in}}$ represents the volume of the specified ROI (*e.g.,* CTV or PTV) that is covered by the chosen isodose, and $V_{\mathrm{ID}}^{\mathrm{total}}$ is the total isodose volume. Conformity was measured using isodose volumes with 80, 90, 95, and 98 % of the prescription dose.

Conformity and DVH criteria (sec. 2.3.3) were compared between ATP and ATS using the non-parametric Wilcoxon signed-rank test [19]. Calculated p-values were corrected using the Benjamini-Hochberg method.

#### 2.3.4 Quantification of variation in daily patient anatomy

Interfractional variations in the rectum and bladder were quantified as the relative position change from the reference image. The volume overlap between these OARs and a set of uniformly expanded volumes from the PTV (0, 5, 10, 15 mm expansions) was calculated on reference and localization images (Supplemental Fig. S2). Changes in overlap between the reference and daily images (ΔPTV) were compared to DVH differences between ATP and ATS using Pearson correlation [20].

## 3 Results

### 3.1 Comparison of fractional and accumulated DVH metrics

**Fig. 2:**
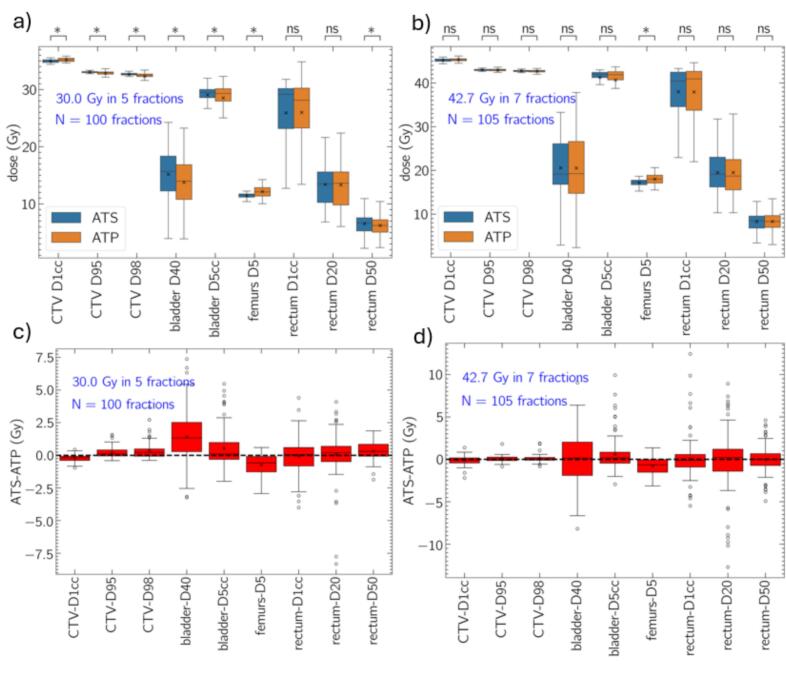
Fractional dose metrics (30.0/5:a, 42.7/7:b) in addition to patient-wise difference (30.0/5:c, 42.7/7:d) patients undergoing. The box extends between the upper and lower quartiles, mean and median are represented by the x and horizonal line. For patient-wise difference plots (c,d) outliers are presented. The p-value notation presented represents the following: * p < 0.05, ns p > 0.05.

**Fig. 3:**
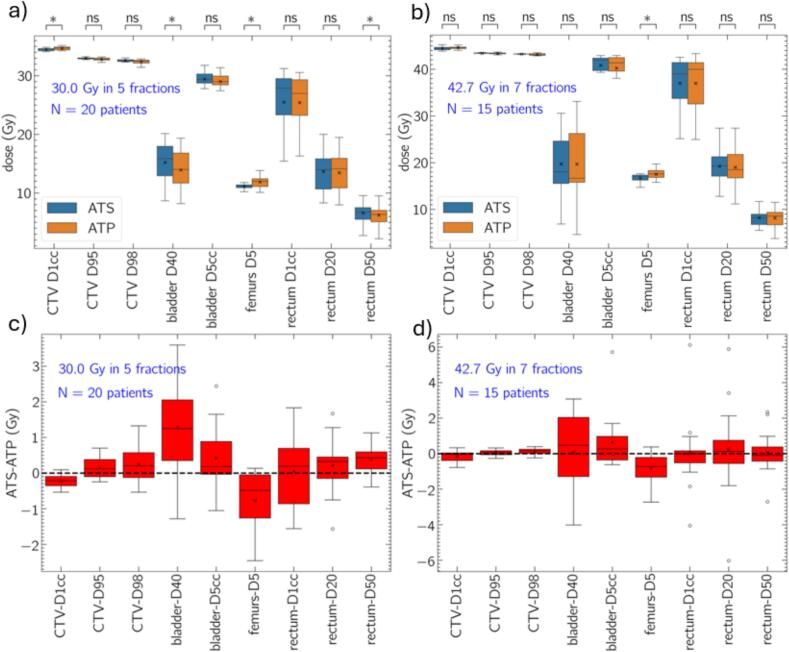
Accumulated dose metrics (a,b) and patient-wise difference (c,d) respectively. Data are presented for 30.0/5 (a,c) and 42.7/7 (b,d). Fractional dose data are normalized based on the inverse of total the number of fractions delivered. The box extends between the upper and lower quartiles, mean and median are represented by the x and horizonal line. For patient-wise difference plots (c,d) outliers are presented. The p-value notation presented represents the following: * p < 0.05, ns p > 0.05.

Fig. 2, Fig. 3 present fractional and accumulated dosimetric comparisons of the ATS and ATP workflows, respectively. Fractional ATS plans demonstrated improved target coverage and generally higher dose to targets and OARs compared to ATP for the 30.0/5 cohort. CTV D98 and D95, bladder D5cc and D40, and rectum D50 were all statistically higher for ATS compared to ATP, although difference magnitudes were small (average difference for each measure: within 1.4 Gy or 5 % of the prescription dose). For patients treated with 42.7/7, similar trends were observed but differences in target and OAR DVH criteria were statistically equivalent, except lower Femur D5 for ATS.

For total accumulated dose, CTV D95 and D98, bladder D5cc, rectum D1cc and D20 were statistically equivalent for the 30.0/5 cohort and average differences were generally small in magnitude (within 0.42 Gy or 1.4 % of the prescription dose for all metrics). Similar trends were observed in the 42.7/7 cohort, except higher Femurs D5 for ATP.

Patient-wise differences in fractional and accumulated dose metrics are presented in Fig. 2(c, d) and Fig. 3(c, d), respectively. For fractional data, large positive and negative differences are observed for some fractions indicating that neither ATS nor ATP is generally dosimetrically superior. For example, positive and negative differences in ATS-ATP exceeding 5.0 Gy are observed for fractional values of rectum D20 for both 30.0/5 and 42.7/7 data. Differences between ATS and ATP are generally larger for fractional doses than for accumulated doses.

### 3.2 Clinical goal pass rates

Supplemental Table S2 presents the percentage of patients failing clinical goals for both fractional and accumulated DVH criteria. For fractional clinical goals, large (>10 %) differences in the failure rate were observed between ATS and ATP for some clinical goals. For example, ATP failed more frequently for CTV D2cc (54 % vs. 34 %) for the 30.0/5 cohort, but not the 42.7/7 cohort (0 % vs 0 %). Failure rates for ATS and ATP strategies are generally lower for accumulated dose than fractional dose, but similar trends between ATS and ATP were observed.

### 3.3 Dose conformity

**Fig. 4:**
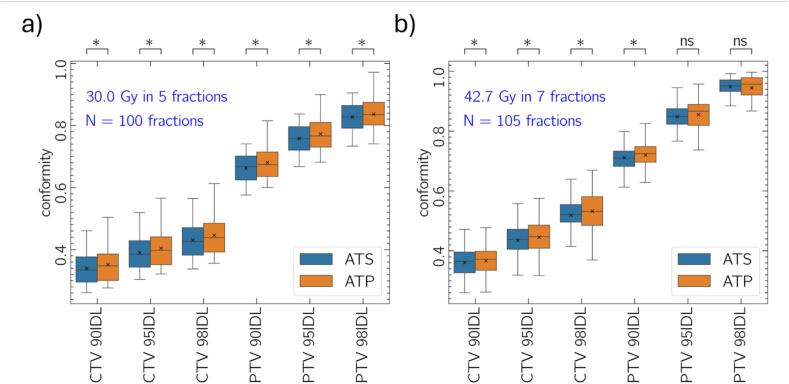
Target conformity (Equation (1) measured for CTV and PTV volumes, relative to *iso*-dose volumes relative to 90, 95 and 98 % of the prescription dose for patients undergoing 30.0/5 (a) and 42.7/7 (b). The box extends between the upper and lower quartiles, mean and median are represented by the x and horizonal line. The p-value notation presented represents the following: * p < 0.05, ns p > 0.05.

Fig. 4 presents dose conformity for fractional and accumulated dose across both 30.0/5 and 42.7/7 treatments. ATP plans are more conformal to both the CTV and PTV.

### 3.4 Relationship between daily patient anatomy changes and adaptive workflows

**Fig. 5:**
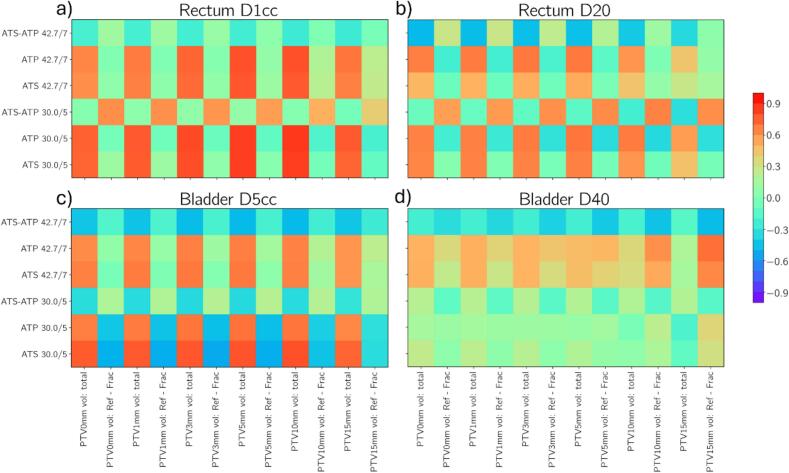
Correlation matrices for patients undergoing 30.0/5 and 42.7/7 which relate fractional dosimetric measures (rectum D1cc; a, rectum D20; b, bladder D5cc; c, and bladder D40; d) from ATS and ATP based plans in conjunction with the difference between respective dosimetic measures (ATS-ATP) to geometric measures for the respective organs presented.

Fig. 5 presents correlation matrices relating rectum and bladder dose metrics to the interfraction organ motion in terms of overlap with expanded PTV volumes. Differences in rectum D1cc moderately correlate with changes in rectum-PTV overlap for patients in the 30.0/5 cohort (maximum of R = 0.62 when using the zero mm PTV expansion to quantify overlap). That is, interfraction motion of the rectum towards the target (increasing overlap with the PTV expansions) are associated with an ATP advantage (higher ATS dose compared to ATP). A similar correlation is not observed for the 42.7/7 cohort. Depending on the OAR and DVH metric considered, the degree of correlation depends on the size of the PTV expansion used to quantify overlap. For example, for patients undergoing 42.7/7, changes in bladder D40 are more strongly correlated with changes in the bladder-PTV overlap for 15 mm PTV expansion (R = −0.46) in comparison to the original PTV volume with zero expansion (R = −0.22).

## 4 Discussion

Maximizing the dosimetric benefits of ART requires robust evaluation of adaptation strategies and assessment of total delivered dose over the treatment course. On the Elekta Unity platform, which necessitates daily adaptation, dose accumulation via deformable image registration (DIR) is essential due to the absence of a traditional reference plan and continuous modification of daily delivery.

In this MR-guided prostate ART study, comparing ATP and ATS revealed small statistical differences in accumulated OAR doses—none likely clinically significant (maximum mean differences < 1.28 Gy or 4.3 % for 30.0/5; <0.83 Gy or 2.0 % for 42.7/7). Some fractional dose metrics reached statistical significance but largely diminished upon accumulation, supporting the idea that voxel-level variations average out over the course (Section 3.1). Clinical objective failures were more frequent in fractional than accumulated analyses (Section 3.2), underscoring the value of dose accumulation in ART evaluation. Previous work comparing online adaptive MR guidance versus conventional image-guided radiation therapy with cone-beam CT demonstrated small differences in accumulated dose for matched PTV margins [21] like our study, but large differences when larger PTV margins were employed for the conventional IGRT [22].

The ATP vs. ATS comparison highlights the importance of interfraction anatomical changes. ATP compensates for setup shifts through isocenter translation and requires reproducible positioning but cannot handle patient rotation or deformation. ATS addresses these through recontouring and full reoptimization (Supplemental Fig. S3).

Despite similar accumulated doses, substantial per-fraction differences in OAR doses were observed between ATP and ATS (Fig. 2c,d). In some instances, ATP achieved superior OAR sparing; in others, ATS performed better. These variations appear related to how each method responds to interfraction organ motion. For example, rectal displacement can result in favorable or unfavorable dosimetric conditions depending on whether the rectum moves toward or away from the prostate (Supplemental Fig. S3). When the rectum moves further away (decreasing PTV overlap), rectum optimization terms in the ATS objective function may yield lower values/penalties than in the reference plan, generating less-aggressive sparing compared to ATP. Conversely, as the rectum moves closer to the target, rectum-sparing objectives for ATS may increase in value/penalty, generating more aggressive sparing than ATP. We hypothesize the use of ATS optimization objectives that are copied from the reference plan, but minimally modified due to limited time, may result in less OAR sparing compared to ATP for some fractions where the anatomy is more favorable. This could be explained by some OAR objective values (*e.g.* for max dose) decreasing in magnitude and gradient if the OAR is further from the target, thereby relaxing the conformality of the reoptimized ATS dose. These results may inform online optimization strategy improvements such as introduction of automated planning [23].

ATS theoretically offers advantages in cases with large anatomical deviations; however, such events were rare in our cohort and had minimal influence on cumulative dose. Even in ultra-hypofractionated regimens, these benefits tended to average out. Notably, ATS plans demonstrated reduced conformality compared to ATP, both fractionally and cumulatively, which may reflect trade-offs in the online ATS optimization process where target and OAR doses are prioritized over conformality.

Our findings suggest no single strategy is optimal for all patients. ATS's resource burden does not yield clinically meaningful benefit across the board. Rather, tailoring the strategy per fraction, based on specific patient features, may optimize target coverage and OAR sparing, achieving the best therapeutic ratio with online ART [14,21].

Recent studies have proposed methods for *a priori* selection of adaptation strategy, such as explicit examination of the deformation vector fields generated from DIR between reference and daily images [4,24,25]. The post-hoc geometric overlap analysis in this study yielded simple explainable metrics that may offer the potential for decision support models that select the optimal ART strategy. Although the anatomical features investigated in this work rely on OAR segmentation on the daily images, automated contouring using machine learning could support this task without increasing resource demands [26,27].

Interesting possibilities remain for future investigations based on this work. For example, MR-guidance has motivated recent margin reduction in the treatment of SBRT [22,28]. Although the results presented herein are specific to the disease site, planning technique, PTV margin and dose fractionation considered, the dose accumulation and analysis framework which was developed and presented may be used for different investigations. For example, the workflow may be used to examine different treatment sites and techniques or as a needed technical step to improve understandings of clinically relevant endpoints [29]. Previous work demonstrates that evaluation of accumulated dose for outcome analysis improves the statistical power of modelling predictions across multiple clinical sites [30,31] including prostate [21,32]. Integration of outcome prediction with the dose accumulation data presented herein is a natural progression of the work presented. Similarly, building accompanying radiobiological effects associated with the varying fractional exposure is an interesting avenue for future research.

The DIR has limitations in the uncertainty of the accumulated dose. The DIR algorithm models anatomical differences using a smooth deformation vector field that is continuous across organ boundaries, but it does not explicitly account for sliding interfaces, such as the prostate moving against the rectum or bladder. This approach assumes consistent contact between the rectum and bladder walls with the prostate in each fraction, potentially overestimating the volume of these organs receiving high doses (impacting metrics like D1cc) while underestimating intermediate dose volumes (affecting D40). The dose warping method used in this study transforms dose distributions between images using the deformation vector field under the assumption of mass conservation, which is inaccurate for rectal and bladder contents but may be valid for their walls, which is of greater radiobiological relevance. To enhance robustness against DIR and dose accumulation uncertainties, this study compares both fractional and accumulated doses. While fractional dose captures daily anatomical variations in plan quality, accumulated dose extends the analysis to identify systematic trends. These datasets are complementary, and by comparing trends in accumulated data with fractional data, the findings remain supported regardless of the underlying DIRs used.

Some limitations in this study relate to both the patient cohort size and the retrospective nature of the comparison. Furthermore, this retrospective comparison does not account for the impact of intrafraction motion throughout the treatment session, from localization to beam delivery, which could affect the robustness of each planning strategy [24,25,33]. Given that the ATP workflow is more time-efficient than ATS, intrafraction motion may have less impact on ATP, potentially leading to a dosimetric advantage that was not considered in this analysis. The retrospective and simulated nature of this investigation did not capture the potential differences across both ATS and ATP deliveries as ATP plans were not clinically treated. To better compare ATP and ATS workflows, a prospective multi-institutional randomized trial could be conducted, comparing reconstructed dose on images acquired during radiation delivery.

## CRediT authorship contribution statement

**Iymad R. Mansour:** Data curation, Investigation, Methodology, Formal analysis. **Chris D. Johnstone:** Data curation. **Victor Malkov:** Conception, Visualization. **Daniel Létourneau:** Visualization. **Peter Chung:** Visualization. **Tony Tadic:** Conceptualization, Methodology, Software, Supervision, Visualization. **Jeff D. Winter:** Conceptualization, Methodology, Software, Supervision, Visualization.

## Declaration of competing interest

The authors declare that they have no known competing financial interests or personal relationships that could have appeared to influence the work reported in this paper.

## References

[1] Winkel D., Bol G.H., Kroon P.S., van Asselen B., Hackett S.S., Werensteijn-Honingh A.M. (2019). Adaptive radiotherapy: the Elekta Unity MR-linac concept. Clin Transl Radiat Oncol.

[2] Acharya S., Fischer-Valuck B.W., Kashani R., Parikh P., Yang D., Zhao T. (2016). Online magnetic resonance image guided adaptive radiation therapy: first clinical applications. Int J Radiat Oncol Biol Phys.

[3] Bohoudi O., Bruynzeel A.M.E., Senan S., Cuijpers J.P., Slotman B.J., Lagerwaard F.J. (2017). Fast and robust online adaptive planning in stereotactic MR-guided adaptive radiation therapy (SMART) for pancreatic cancer. Radiother Oncol.

[4] Xia W.L., Liang B., Men K., Zhang K., Tian Y., Li M.H. (2023). Prediction of adaptive strategies based on deformation vector field features for MR-guided adaptive radiotherapy of prostate cancer. Med Phys.

[5] Winkel D. (2019). Adaptive radiotherapy: the Elekta Unity MR-linac concept. Clin Transl Radiat Oncol.

[6] Murr M., Brock K.K., Fusella M., Hardcastle N., Hussein M., Jameson M.G. (2023). Applicability and usage of dose mapping/accumulation in radiotherapy. Radiother Oncol.

[7] Chetty I.J., Rosu-Bubulac M. (2019). Deformable registration for dose accumulation. Semin Radiat Oncol.

[8] Chen J., Bissonnette J.-P., Craig T., Munoz-Schuffenegger P., Tadic T., Dawson L.A. (2023). Liver SBRT dose accumulation to assess the impact of anatomic variations on normal tissue doses and toxicity in patients treated with concurrent sorafenib. Radiother Oncol.

[9] Bohoudi O., Bruynzeel A.M.E., Tetar S., Slotman B.J., Palacios A., Lagerwaard F.J. (2021). Dose accumulation for personalized stereotactic MR-guided adaptive radiation therapy in prostate cancer. Radiother Oncol.

[10] Bosma L., Zachiu C., Ries M., Senneville B.D., Raaymakers B. (2021). Quantitative investigation of dose accumulation errors from intra-fraction motion in MRfRT for prostate cancer. Phys Med Biol.

[11] Wollenberg W., Carbaat C., Ruiter P., Remeijer P., Janssen T., Sonke J. (2019). EP-2004 Online rotation correction for MR-guided prostate radiotherapy. Radiother Oncol.

[12] Kontaxis C. (2020). Delivered dose quantification in prostate radiotherapy using online 3D cine imaging and treatment log files on a combined 1.5T magnetic resonance imaging and linear accelerator system. Phys Imaging Radiat Oncol.

[13] Yang J. (2021). Online adaptive planning for prostate stereotactic body radiotherapy using a 1.5 Tesla magnetic resonance imaging-guided linear accelerator. Phys Imaging Radiat Oncol.

[14] Winkel D. Online treatment adaptation strategies for the 1.5T MR-linac: first implementation and evaluation for lymph node oligometastases. n.d.

[15] Oh S., Kim S. (2017). Deformable image registration in radiation therapy. Radiat Oncol J.

[16] García-Mollá R., de Marco-Blancas N., Bonaque J., Vidueira L., López-Tarjuelo J., Perez-Calatayud J. (2015). Validation of a deformable image registration produced by a commercial treatment planning system in head and neck. Phys Med.

[17] Malkov V., Winter J.D., Kong V., Li W., Dang J., Navarro I. (2022). OC-0953 Dosimetric validation of a hybrid DIR algorithm for MR-Linac dose accumulation. Radiother Oncol.

[18] Malkov VN, Mansour IR, Kong V, Li W, Dang J, Sadeghi P, Navarro I, Padayachee J, Chung P, Winter JD, Tadic T. Geometric and dosimetric validation of deformable image registration for prostate MR-guided adaptive radiotherapy 2025;2504.07933. https://doi.org/https://arxiv.org/abs/2504.07933.

[19] Virtanen P., Gommers R., Oliphant T.E., Haberland M., Reddy T., Cournapeau D., Burovski E., Peterson P., Weckesser W., Bright J., van der Walt S.J., Brett M., Wilson J., Millman K.J., Mayorov N., Nelson A.R.J., Jones E., Kern R., Larson E., Carey C.J., Polat İ., Feng Y., Moore E.W., VanderPlas J., Laxalde D., Perktold J., Cimrman R., Henriksen I., Quintero E.A., Harris C.R., Archibald A.M., Ribeiro A.H., Pedregosa F., van Mulbregt P., Vijaykumar A., Pietro B.A., Rothberg A., Hilboll A., Kloeckner A., Scopatz A., Lee A., Rokem A., Woods C.N., Fulton C., Masson C., Häggström C., Fitzgerald C., Nicholson D.A., Hagen D.R., Pasechnik D.V., Olivetti E., Martin E., Wieser E., Silva F., Lenders F., Wilhelm F., Young G., Price G.A., Ingold G.-L., Allen G.E., Lee G.R., Audren H., Probst I., Dietrich J.P., Silterra J., Webber J.T., Slavič J., Nothman J., Buchner J., Kulick J., Schönberger J.L., de Miranda Cardoso J.V., Reimer J., Harrington J., Rodríguez J.L.C., Nunez-Iglesias J., Kuczynski J., Tritz K., Thoma M., Newville M., Kümmerer M., Bolingbroke M., Tartre M., Pak M., Smith N.J., Nowaczyk N., Shebanov N., Pavlyk O., Brodtkorb P.A., Lee P., McGibbon R.T., Feldbauer R., Lewis S., Tygier S., Sievert S., Vigna S., Peterson S., More S., Pudlik T., Oshima T., Pingel T.J., Robitaille T.P., Spura T., Jones T.R., Cera T., Leslie T., Zito T., Krauss T., Upadhyay U., Halchenko Y.O., Vázquez-Baeza Y. (2020). Contributors S 1. 0. SciPy 1.0: fundamental algorithms for scientific computing in Python. Nat Methods.

[20] McKinney W. Data Structures for Statistical Computing in Python. Proceedings of the 9th Python in Science Conference 2010;56–61:51–6. https://doi.org/https://doi.org/10.25080/Majora-92bf1922-00a.

[21] Murr M., Wegener D., Böke S., Gani C., Mönnich D., Niyazi M. (2024). Comparison of online adaptive and non-adaptive magnetic resonance image-guided radiation therapy in prostate cancer using dose accumulation. Phys Imaging Radiat Oncol.

[22] Christiansen R.L., Dysager L., Hansen C.R., Jensen H.R., Schytte T., Nyborg C.J. (2022). Online adaptive radiotherapy potentially reduces toxicity for high-risk prostate cancer treatment. Radiother Oncol.

[23] Khalifa A., Winter J.D., Tadic T., Purdie T.G., McIntosh C. (2024). Machine learning automated treatment planning for online magnetic resonance guided adaptive radiotherapy of prostate cancer. Phys Imaging Radiat Oncol.

[24] Lim S.N., Ahunbay E.E., Nasief H., Zheng C., Lawton C., Li X.A. (2020). Indications of online adaptive replanning based on organ deformation. Pract Radiat Oncol.

[25] Parchur A.K., Lim S., Nasief H.G., Omari E.A., Zhang Y., Paulson E.S. (2023). Auto-detection of necessity for MRI-guided online adaptive replanning using a machine learning classifier. Med Phys.

[26] Baroudi H., Brock K.K., Cao W., Chen X., Chung C., Court L.E. (2023). Automated contouring and planning in radiation therapy: what is ‘clinically acceptable’?. Diagnostics.

[27] Kawamura M., Kamomae T., Yanagawa M., Kamagata K., Fujita S., Ueda D. (2024). Revolutionizing radiation therapy: the role of AI in clinical practice. J Radiat Res.

[28] Kishan A.U., Ma T.M., Lamb J.M., Casado M., Wilhalme H., Low D.A. (2023). Magnetic resonance imaging–guided vs computed tomography–guided stereotactic body radiotherapy for prostate cancer: the MIRAGE randomized clinical trial. JAMA Oncol.

[29] Jaffray D.A., Lindsay P.E., Brock K.K., Deasy J.O., Tomé W.A. (2010). Accurate accumulation of dose for improved understanding of radiation effects in normal tissue. Int J Radiat Oncol Biol Phys.

[30] McCulloch M.M., Muenz D.G., Schipper M.J., Velec M., Dawson L.A., Brock K.K. (2018). A simulation study to assess the potential impact of developing normal tissue complication probability models with accumulated dose. Adv Radiat Oncol.

[31] Swaminath A., Massey C., Brierley J.D., Dinniwell R., Wong R., Kim J.J. (2015). Accumulated delivered dose response of stereotactic body radiation therapy for liver metastases. Int J Radiat Oncol Biol Phys.

[32] Shelley L.E.A., Scaife J.E., Romanchikova M., Harrison K., Forman J.R., Bates A.M. (2017). Delivered dose can be a better predictor of rectal toxicity than planned dose in prostate radiotherapy. Radiother Oncol.

[33] Dassen M.G., Janssen T., Kusters M., Pos F., Kerkmeijer L.G.W., van der Heide U.A. (2023). Comparing adaptation strategies in MRI-guided online adaptive radiotherapy for prostate cancer: Implications for treatment margins. Radiother Oncol.

