# Supplementary material for: Comparison of online adaptation strategies for magnetic resonance guided prostate radiation therapy
Source: Phys Imaging Radiat Oncol. 2025 Jul 25;35:100816. doi: 10.1016/j.phro.2025.100816 (PMC12361782; doi:10.1016/j.phro.2025.100816)
Supplement: Supplementary Data 1 [file mmc1.docx]

Supplementary figures and tables

| **ROI** | **Metric** | **3000/5 (cGy)** | **4270/7 (cGy)** |
| --- | --- | --- | --- |
| CTV | D99 | - | >4270 |
|  | D95 | >3300 | - |
| PTV | D99 | - | >4060 |
|  | D98 | >2850 | - |
|  | D95 | >3000 | - |
|  | D2 | <3500 | - |
|  | D1cc | - | <4610 |
|  | D0cc | <4000 | - |
| rectum | D20 | <2000 | <2400 |
|  | D50 | <1000 | <1000 |
| bladder | D5cc | <3000 | <4270 |
|  | D40 | <1500 | <1800 |
| femur  (left or right) | D5 | <1200 | <1800 |
| penile bulb | D1cc | <3000 | <4270 |
|  | D50 | <2400 | <3420 |
| small bowel | D1cc | < 2500 | <3000 |
|  | D5cc | <1500 | - |
| large bowel | D1cc | <2500 | <3000 |
|  | D5cc | <1500 | - |
| Urethra | D50 | <3500 | <4500 |

Supplemental Table S1: Target and organ-at-risk clinical planning goals for 3000 cGy in 5 fractions (3000/5) and 4270 cGy in 7 fractions (4270/7).

|  |  | **3000/5** | | | | **4270/7** | | | |
| --- | --- | --- | --- | --- | --- | --- | --- | --- | --- |
|  |  | Fractional | | Accumulated | | Fractional | | Accumulated | |
| ROI | Metric | ATS (%) | ATP (%) | ATS (%) | ATP (%) | ATS (%) | ATP (%) | ATS (%) | ATP (%) |
| CTV | D98 | 0 | 0 | 0 | 0 | 0 | 0 | 0 | 0 |
|  | D95 | 0 | 0 | 0 | 0 | 0 | 0 | 0 | 0 |
|  | D2cc | 34 | 54 | 0 | 20 | 0 | 0 | 0 | 0 |
| Rectum | D1cc | 29 | 31 | 15 | 20 | 11 | 25 | 0 | 13 |
|  | D20 | 1 | 5 | 5 | 5 | 13 | 18 | 7 | 13 |
|  | D50 | 1 | 1 | 0 | 0 | 18 | 22 | 20 | 20 |
| Bladder | D5cc | 20 | 28 | 35 | 20 | 10 | 17 | 7 | 7 |
|  | D40 | 48 | 36 | 55 | 45 | 61 | 57 | 53 | 47 |
| Femurs | D5 | 4 | 56 | 0 | 50 | 10 | 52 | 0 | 33 |

Supplemental Table S2: the percentage of either 3000/5 or 4270/7 patients failing clinical goals (Supplemental Table S1) for both fractional and accumulated data.


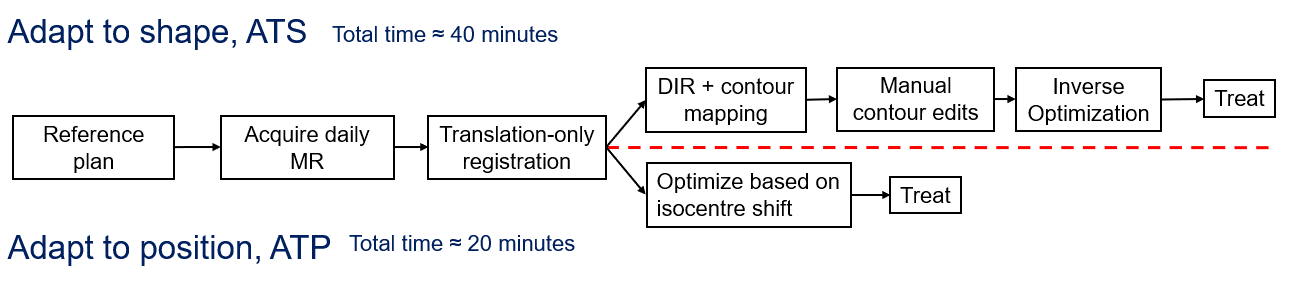


Supplemental Figure S1: Adapt to shape (ATS) and Adapt to position (ATP) workflows available for the Elekta Unity Magnetic Resonance Linac.


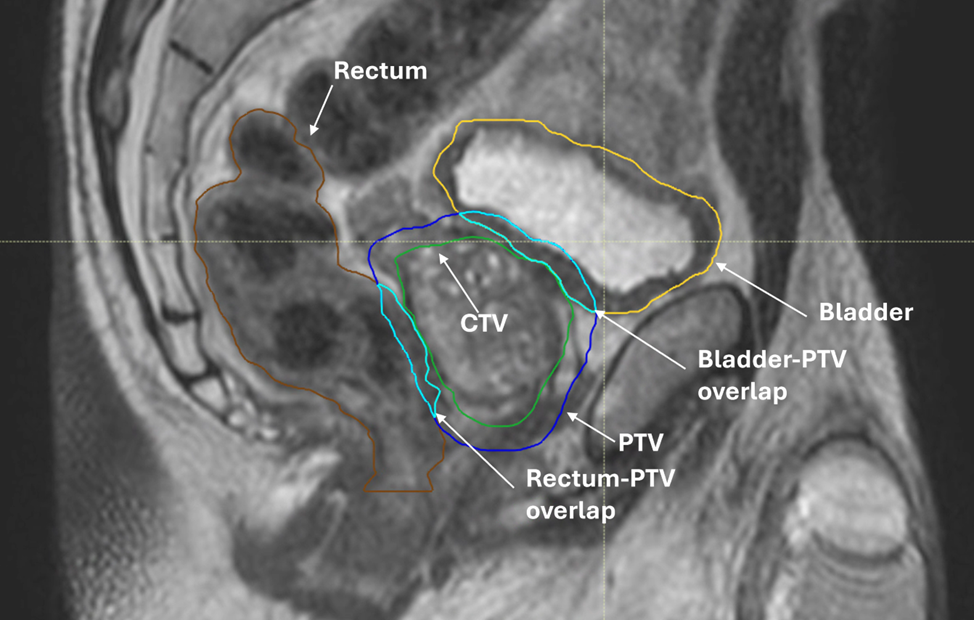


Supplemental Figure S2: In the example shown, the bladder (yellow), rectum (brown), CTV (green) and PTV (blue) volumes are presented. The volumes in which the rectum and bladder respectively overlap with the PTV are presented (cyan).

| a)  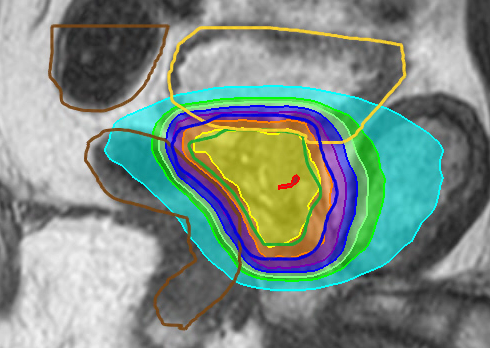 | b)  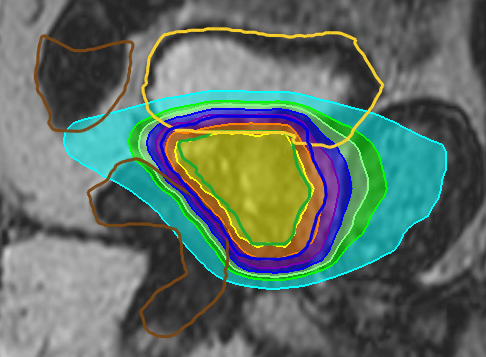 | c)  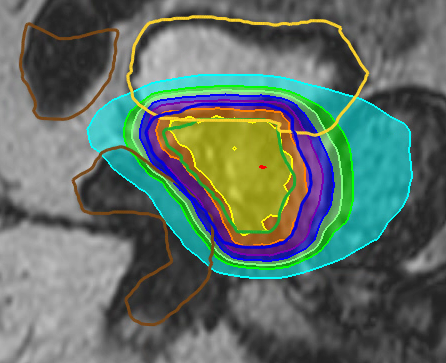 | 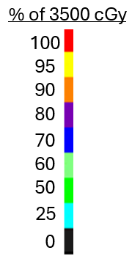 |
| --- | --- | --- | --- |
| d)  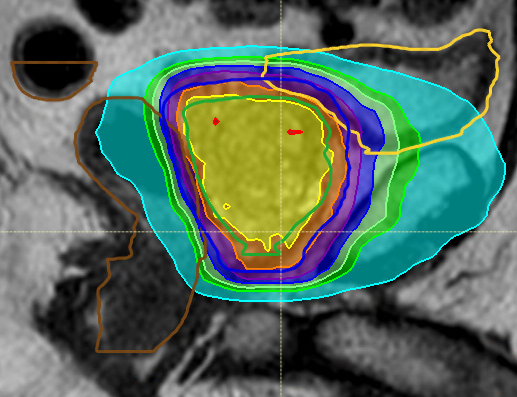 | e)  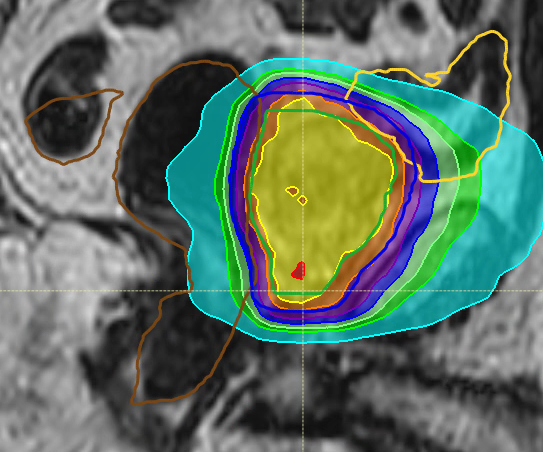 | f)  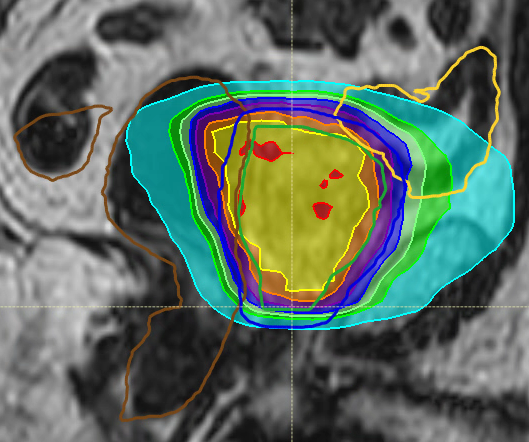 |  |

Supplemental Figure S3: Examination of variation in rectum position between reference and daily images for a patient whose rectum moves closer to the target volume due to gas (a; reference, b; ATS, c; ATP and another patient whose rectum moves away from target volume (d; reference, e; ATS, f; ATP).
